# The impact of nutritional intervention on the prognosis of PCOS patients with different BMIs

**DOI:** 10.3389/fmed.2026.1650724

**Published:** 2026-03-11

**Authors:** Shujun Ma, Wanliang Chen

**Affiliations:** 1 Reproductive Health Department, Xiantao Maternity and Child Healthcare Hospital, Xiantao, China; 2 Hubei Cancer Hospital, Tongji Medical College, Huazhong University of Science and Technology, Wuhan, China

**Keywords:** body mass index, interaction, nutritional intervention, ovulation rate, polycystic ovary syndrome

## Abstract

**Background and objectives:**

Polycystic ovary syndrome (PCOS) is a common endocrine disorder in women, often associated with metabolic abnormalities and ovulatory dysfunction. This study aimed to assess the effect of body mass index (BMI) and its interactions on nutritional intervention outcomes in PCOS patients.

**Methods and study design:**

A total of 360 PCOS patients were retrospectively analyzed. Ovulation rates and metabolic indices before and after treatment were compared across BMI groups. A multivariate regression analysis was used to assess the influence of BMI, age, symptom duration, family history, and ovarian enlargement on intervention outcomes. Interaction effects between BMI and other variables were also examined.

**Results:**

After treatment, ovulation rates, homeostasis model assessment of insulin resistance (HOMA-IR), low-density lipoprotein (LDL), high-density lipoprotein (HDL), and fasting plasma glucose (FPG) improved significantly in all groups, with the obese group showing the greatest BMI reduction (−5.8%) and most favorable response. A multivariate analysis indicated that poorer outcomes were associated with older age (odds ratio (OR) = 0.993), longer symptom duration (OR = 0.982), a family history of PCOS (OR = 0.745), ovarian enlargement (OR = 0.887), and a higher number of ovarian cysts (OR = 0.882). Conversely, higher BMI (OR = 1.089) and HDL (OR = 1.010) were associated with better outcomes. Interaction analysis revealed that age attenuated the positive effect of BMI (OR = 0.992), and ovarian enlargement further diminished BMI’s beneficial impact (OR = 0.759).

**Conclusion:**

Obese patients derived the greatest benefit from nutritional intervention. Higher BMI was associated with better outcomes, particularly among younger patients and those with less ovarian enlargement. These findings support the use of personalized nutrition strategies to enhance treatment efficacy in PCOS management.

## Introduction

Polycystic ovary syndrome (PCOS) is a common endocrine disorder affecting approximately 5–10% of women of reproductive age (1, 2). It is primarily characterized by ovulatory dysfunction and metabolic abnormalities, resulting in symptoms such as infertility, irregular menstruation, and hyperandrogenism. Patients frequently exhibit insulin resistance, dyslipidemia, and metabolic issues including obesity (3, 4). Consequently, women with PCOS face the dual burden of reproductive and metabolic disturbances. These manifestations not only compromise fertility but also increase the long-term risk of chronic conditions such as diabetes and cardiovascular disease (5, 6).

Multiple studies have demonstrated a close association between obesity and PCOS (7). Obesity can exacerbate the metabolic disturbances observed in PCOS, leading to insulin resistance, hyperglycemia, and dyslipidemia, thereby creating a vicious cycle (8–10). Consequently, weight management and nutritional interventions have become essential components of PCOS treatment. Evidence suggests that scientifically guided dietary modifications and effective weight control can significantly improve insulin sensitivity, blood glucose, and lipid profiles in PCOS patients, helping to restore ovulatory function and enhance fertility (11, 12). Beyond metabolic improvements, weight management also contributes to overall health, reducing the risk of complications. Therefore, personalized weight management and nutrition programs are pivotal in the comprehensive care of PCOS, effectively improving metabolic health and supporting reproductive recovery (13).

At present, research on nutritional intervention for PCOS patients is limited (14); however, there is still relatively little research on the role of body mass index (BMI) and its interaction in nutritional intervention for PCOS (14). Understanding the complex relationships between these factors is of great significance for precision treatment. This study retrospectively analyzed the treatment outcomes of PCOS patients in different BMI groups, combined with multivariate regression and interaction analyses, to explore the combined effects of BMI and other factors on treatment outcomes.

## Materials and methods

### Research subject

This retrospective study included 360 patients with PCOS, diagnosed according to the Rotterdam criteria (15), who were treated at our hospital from January 2021 to January 2024. All participants signed informed consent forms. Patients were divided into three groups based on BMI: normal weight group (BMI 18.5–24.9), overweight group (BMI 25–29.9), and obese group (BMI > 30) (16). The exclusion criteria were as follows: (1) patients with other reproductive system diseases and endocrine disorders, such as thyroid dysfunction (hyperthyroidism or hypothyroidism), hyperprolactinemia, and Cushing’s syndrome; (2) pregnant or lactating women; (3) severe liver and kidney dysfunction; and (4) patients with mental or cognitive impairments. This study was approved by the Ethics Committee of Hubei Cancer Hospital, Tongji Medical College, and Huazhong University of Science and Technology and conducted in accordance with the Declaration of Helsinki. All participants provided written informed consent. Using a medium effect size, a significance level of 0.05, and a statistical power of 80%, for continuous variables, the required sample size was at least 53 participants per group. For categorical variables, the total required sample size was 240, with an average of 80 participants per group. The sample size in this study meets these requirements. A total of 412 PCOS patients were initially enrolled in the study. After the intervention and follow-up, 360 patients completed the full intervention and follow-up, with 52 dropouts, corresponding to a dropout rate of approximately 12.6%. The primary reasons for dropout included loss to follow-up, incomplete adherence to the dietary plan, or personal reasons.

### Nutritional intervention

The nutritional intervention in this study consisted of comprehensive dietary counseling and a personalized nutrition plan. Each patient received guidance from a professional nutritionist, including the establishment of healthy eating habits, food selection, knowledge of nutritional components, and strategies to maintain a balanced diet in daily life. The intervention emphasized a reasonable distribution of macronutrients: protein accounted for approximately 15–20% of total energy, carbohydrates 40–55%, and fats 25–30%. For PCOS patients with normal weight, the total calorie intake is controlled at 1,200–1,500 kcal/day, while for overweight or obese patients, the calorie intake is 10–20% lower than that of normal patients. The protein intake was set at 1.0–1.2 g/kg/body weight per day and carbohydrate intake was prescribed at 225–325 g/day, with overweight patients consuming approximately 180–225 g/day and obese patients consuming approximately 120–160 g/day. The specific energy intake for each patient was individualized according to their BMR, ensuring that total caloric intake was slightly above BMR to meet basic metabolic needs. Carbohydrate intake was recommended to account for 40–55% of total energy requirements and was further adjusted based on patients’ clinical indicators (e.g., insulin resistance index, fasting plasma glucose, and lipid profile). This approach ensured nutritional balance while maximizing the effectiveness of the intervention. The intervention lasted for 3 months.

With regard to dietary quality, this study emphasized the rational selection of nutrient sources and food choices. Carbohydrates were primarily derived from low-glycemic index (GI) and high-fiber foods, such as whole grains, legumes, vegetables, and fruits, while the intake of refined grains (e.g., white rice and white flour) and high-sugar foods was reduced. Protein intake focused on high-quality sources, including fish, poultry, eggs, legumes, dairy products, and nuts, with limited consumption of processed meats. Fat sources were mainly unsaturated fatty acids, derived from olive oil, fish oil, nuts, and avocados, while intake of saturated and trans fats was minimized. This approach not only ensured appropriate macronutrient distribution but also optimized dietary quality, thereby improving insulin sensitivity and metabolic outcomes.

In this study, a 3-day food record was used to document patients’ dietary intake. Participants were instructed to consecutively record all foods and beverages consumed over 3 days (including two weekdays and one weekend day) within each 2-week follow-up period, detailing food types, portion sizes, and cooking methods. Dietary compliance was assessed based on the degree of deviation between actual dietary intake and the prescribed nutritional targets. During the 3-month intervention, patients were managed through a combination of regular follow-ups and daily monitoring. Patients attended clinic visits every 2 weeks, during which dietitians evaluated dietary compliance and physical indicators and modified dietary plans as necessary. In addition, these data were submitted to the research team through an online platform or telephone to support process management and compliance monitoring.

### Data collection

Baseline information of different BMI patients was collected, including age, duration of symptoms (years), family history of PCOS, cardiovascular disease and diabetes, and regularity of menstrual cycle (regular, irregular, and no menstruation) (17). The duration of a menstrual cycle may be classified as normal (21–35 days), brief (less than 21 days), or longer (exceeding 35 days). Menstrual flow can be divided into normal (30–80 mL, change sanitary pads or tampons every 4–6 h), excessive (more than 80 mL, change sanitary pads or tampons every 2 h or less, and last for 7 days), or insufficient (less than 30 m, change sanitary pads or tampons every 6 h or more) (18). The degree of ovarian enlargement was classified as normal (2–4 cm), mild enlargement (4–5 cm), and severe enlargement (>5 cm). The number of follicles was classified as normal (8–11), mildly increased (12–20), and significantly increased (>20) (19), while ovarian cysts are classified as no cysts (0–1), mild cysts (2–3), and severe cysts (>3). Clinical outcomes included ovulation rate, insulin resistance index (HOMA-IR), low-density lipoprotein (LDL) levels, high-density lipoprotein (HDL) levels, fast plasma glucose (FPG) levels, and BMI changes before and after nutritional intervention. After 6 months of treatment, the prognosis was defined as follows: ovulation rate reaching more than 65% (with ovulation occurring for 4 months or more), menstrual cycle being normal or close to normal, BMI decreasing by more than 5% (20), and fasting blood glucose levels decreasing by more than 10% (21). In this study, such outcomes were defined as an improved prognosis; otherwise, prognosis was considered poor.

### Statistical analysis

Continuous data were represented as median (minimum–maximum) and categorical data were expressed as frequency (percentage). A multivariate regression analysis was used to evaluate the impact of factors such as BMI, age, duration of symptoms, family history of PCOS, and degree of ovarian enlargement on the effectiveness of nutritional interventions (with improved prognosis coded as 1 and poor prognosis coded as 0). Interaction terms and ROC curve analysis in the regression model were used to assess the impact of the interaction between BMI and other variables on the intervention outcomes. All statistical analyses were conducted using R software, and a *p*-value of <0.05 was considered statistically significant.

## Results

### Baseline information of PCOS patients

The age of PCOS patients ranged from 18 to 42 years, with a median age of 29 years, and the median duration of symptoms was 5 years. Regarding menstrual characteristics, the majority of patients presented with an irregular menstruation pattern or prolonged cycles, whereas regular or shortened cycles were relatively less common. More than half of the patients experienced a heavy menstrual flow. Ultrasound findings revealed that the majority of patients had mild ovarian enlargement with 12–20 follicles, although a considerable proportion exhibited severe ovarian enlargement or more than 20 follicles. In addition, more than 90% of patients had multiple ovarian cysts (Table 1). Among the 52 patients who dropped out, 25 were in the normal weight group, 15 in the overweight group, and 12 in the obese group, with comparable proportions across BMI categories. The age distribution was similar among the groups, as was the duration of symptoms. In addition, no significant differences were observed among the groups with respect to menstrual cycle patterns, including regular, irregular, and absent menstruation cycles (Supplementary Table 1).

**Table 1:** Baseline characteristics of PCOS patients with different BMI.

Variables	All patients (*n* = 360)	Normal weight group (*n* = 170)	Overweight group (*n* = 110)	Obese group (*n* = 80)	*p* value
Age	29 (18–42)	29 (18–42)	30 (18–42)	31 (18–42)	0.293
Duration of symptoms (year)	5 (0–8)	4 (0–8)	5 (0–8)	5 (0–8)	0.305
Family history of PCOS					0.340
Yes	36 (10%)	17 (10%)	8 (7.27%)	11 (13.75%)	
No	324 (90%)	153 (90%)	102 (92.73%)	69 (86.25%)	
Cardiovascular disease					0.052
Yes	44 (12.22%)	14 (8.24%)	15 (13.64%)	15 (18.75%)	
No	316 (87.78%)	156 (91.76%)	95 (86.36%)	65 (81.25%)	
Diabetes mellitus					0.135
Yes	45 (12.5%)	15 (8.82%)	17 (15.45%)	13 (16.25%)	
No	315 (87.5%)	155 (91.18%)	93 (84.55%)	67 (83.75%)	
Menstrual cycle regularity					0.056
Regular	101 (28.06%)	43 (25.29%)	31 (28.18%)	27 (33.75%)	
Irregular	219 (60.83%)	114 (67.06%)	66 (60%)	39 (48.75%)	
Absent	40 (11.11%)	13 (7.65%)	13 (11.82%)	14 (17.5%)	
Duration of menstrual period					0.115
Normal	89 (24.72%)	38 (22.35%)	35 (31.82%)	16 (20%)	
Short	42 (11.67%)	19 (11.18%)	16 (14.55%)	7 (8.75%)	
Long	229 (63.61%)	113 (66.47%)	59 (53.64%)	57 (71.25%)	
Menstrual flow					0.094
Normal	90 (25%)	47 (27.65%)	21 (19.09%)	22 (27.5%)	
Heavy	185 (51.39%)	86 (50.59%)	54 (49.09%)	45 (56.25%)	
Light	85 (23.61%)	37 (21.76%)	35 (31.82%)	13 (16.25%)	
Ovarian size					0.382
Normal (2–4 cm)	50 (13.89%)	26 (15.29%)	18 (16.36%)	6 (7.5%)	
Mildly enlarged (4–5 cm)	273 (75.83%)	129 (75.88%)	80 (72.73%)	64 (80%)	
Severely enlarged (>5 cm)	37 (10.28%)	15 (8.82%)	12 (10.91%)	10 (12.5%)	
Number of follicles					0.068
Normal (8–11)	12 (3.33%)	4 (2.35%)	7 (6.36%)	1 (1.25%)	
Mildly increased (12–20)	245 (68.06%)	125 (73.53%)	66 (60%)	54 (67.5%)	
Highly increased (>20)	103 (28.61%)	41 (24.12%)	37 (33.64%)	25 (31.25%)	
Ovarian cysts					0.508
Absent (0–1)	25 (6.94%)	13 (7.65%)	6 (5.45%)	6 (7.5%)	
Mild cysts (2–3)	218 (60.56%)	109 (64.12%)	62 (56.36%)	47 (58.75%)	
Severe cysts (>3)	117 (32.5%)	48 (28.24%)	42 (38.18%)	27 (33.75%)	

PCOS, Polycystic ovary syndrome; BMI, Body mass index.

Statistical tests: The Kruskal–Wallis test was used for continuous variables; the chi-square test or Fisher’s exact test was used for categorical variables.

### Differences in ovulation rate and metabolic indicators before and after nutritional intervention in PCOS patients with different BMI groups

Before treatment, there were no significant differences in the ovulation rate, HOMA-IR, LDL, HDL, and FPG among patients with different BMI groups. After treatment, the ovulation rate significantly improved in all groups (*p* = 0.038), with the obese group showing the most significant improvement. All groups showed a decrease in HOMA-IR (*p =* 0.040), indicating that treatment improved insulin resistance. During the treatment process, the obese group showed the largest change in BMI (−5.8%), and there were significant differences in this change among different BMI groups (*p* = 0.018). After treatment, LDL decreased in all groups (*p* = 0.043), HDL levels increased in all groups (*p* = 0.034), and fasting blood glucose decreased in all groups (*p* = 0.020) (Table 2).

**Table 2:** Treatment-related differences in ovulation and metabolism among different BMI groups.

Variables	All patients (*n* = 360)	Normal weight group (*n* = 170)	Overweight group (*n* = 110)	Obese group (*n* = 80)	*p* value
Ovulation rate					
Before treatment	168 (46.67%)	88 (51.76%)	49 (44.55%)	31 (38.75%)	0.136
After treatment	257 (71.39%)	111 (65.29%)	82 (74.55%)	64 (80%)	0.038
HOMA-IR					
Before treatment	3.1 (2.2–4.1)	3.1 (2.2–4.1)	3.1 (2.2–4.1)	3.3 (2.2–4.1)	0.461
After treatment	1.8 (0.6–2.7)	1.9 (0.6–2.6)	1.6 (0.6–2.7)	1.6 (0.6–2.6)	0.040
BMI change (%)					
	−3.9 (−10.2–2.2)	−3.5 (−10.2–2.1)	−4.0 (−10.2–2.2)	−5.8 (−10.2–1.7)	0.018
LDL (mg/dL)					
Before treatment	119.0 (92.9–142.3)	117.2 (92.9–142.3)	120.4 (92.9–142.3)	121.9 (93.2–142.2)	0.342
After treatment	98.5 (80.6–115.3)	101.4 (80.6–115.3)	98.2 (81.1–114.8)	95.7 (82.0–115.0)	0.043
HDL (mg/dL)					
Before treatment	41.1 (31.6–50.3)	41.3 (31.6–50.3)	41.0 (32.2–50.3)	39.5 (31.6–50.2)	0.767
After treatment	49.4 (38.4–57.8)	49.4 (38.4–57.6)	48.6 (38.4–57.3)	48.1 (39.0–57.8)	0.034
FPG (mmol/L)					
Before treatment	6.6 (4.9–8.1)	6.4 (4.9–8.1)	6.7 (4.9–8.1)	6.6 (4.9–8.1)	0.846
After treatment	5.4 (3.5–7.2)	5.8 (3.5–7.2)	5.2 (3.5–7.1)	5.1 (3.6–7.2)	0.020

BMI, Body mass index; LDL, Low-density lipoprotein; HDL, high-density lipoprotein; FPG, Fasting plasma glucose; HOMA-IR, Homeostasis model assessment of insulin resistance.

Statistical tests: The Kruskal–Wallis test was used for between-group comparisons of continuous variables.

### A multivariate regression analysis of factors affecting the effectiveness of nutritional interventions

The results showed that older age was associated with a slight decrease in intervention effectiveness (odds ratio (OR) = 0.993, *p* = 0.018). The longer the duration of symptoms, the worse the intervention effect (OR = 0.982, *p* = 0.039). Patients with a family history of PCOS showed significantly poorer intervention outcomes (OR = 0.745, *p* < 0.001). Patients with significant ovarian enlargement had poorer intervention outcomes (OR = 0.887, *p* = 0.003). The intervention effect was poor in patients with more ovarian cysts (OR = 0.882, *p* < 0.001). A higher BMI was associated with better intervention outcomes (OR = 1.089, *p* = 0.001). High HDL levels were associated with better intervention outcomes (OR = 1.010, *p* = 0.006) (Table 3).

**Table 3:** Multivariate regression analysis of factors influencing the effectiveness of nutritional intervention.

Term	*B*	Std error	*Z*	*p* value	OR	CI-lower	CI-upper
Age	−0.007	0.003	−2.378	0.018	0.993	0.988	0.999
Duration of symptoms	−0.018	0.009	−2.068	0.039	0.982	0.965	0.999
Family history of PCOS	−0.294	0.067	−4.403	<0.001	0.745	0.654	0.849
Cardiovascular disease	−0.046	0.061	−0.753	0.452	0.955	0.848	1.076
Diabetes mellitus	−0.091	0.060	−1.512	0.131	0.913	0.812	1.027
Ovarian size	−0.120	0.041	−2.946	0.003	0.887	0.819	0.961
Number of follicles	−0.036	0.039	−0.908	0.365	0.965	0.893	1.042
Ovarian cysts	−0.126	0.035	−3.619	<0.001	0.882	0.823	0.944
BMI	0.085	0.026	3.256	0.001	1.089	1.034	1.146
LDL	−0.003	0.001	−1.788	0.075	0.997	0.995	1.000
HDL	0.010	0.004	2.747	0.006	1.010	1.003	1.017

MI, Body mass index; LDL, Low-density lipoprotein cholesterol; HDL, High-density lipoprotein cholesterol; OR, Odds ratio; CI, Confidence interval; *B*: Regression coefficient; Std error: Standard error; *Z*: Wald *Z* statistic.

### Interaction between BMI, age, and degree of ovarian enlargement

The interaction with age indicates that the coefficient is negative (−0.008), which indicates that the relationship between BMI and age had an inhibitory effect on the occurrence of PCOS prognosis improvement. An OR value of 0.992 indicates that, for each additional unit increase of BMI–age combination, the likelihood of PCOS prognosis improvement decreased by approximately 0.8%, indicating thatthe impact of BMI on PCOS prognosis improvement gradually weakened with increasing age. For younger patients, nutritional intervention may exert different effects in patients with different BMI; however, for older patients, the impact of BMI on nutritional intervention becomes less significant. Similarly, the interaction term between BMI and ovarian size was statistically significant (*p* = 0.001), indicating that the combined effect of BMI and ovarian size had an inhibitory effect on the prognosis improvement in PCOS. This suggests that the degree of ovarian enlargement weakened the impact of BMI on nutritional intervention outcomes. In patients with less ovarian enlargement, BMI had a more significant impact on nutritional intervention outcomes, while in patients with more ovarian enlargement, the role of BMI was influenced by other factors such as ovarian dysfunction, thereby weakening its effect (Table 4).

**Table 4:** Interaction of BMI on the effect of nutritional intervention.

Term	*B*	Std error	*Z*	*p* value	OR	CI-lower	CI-upper	OR-Adj	CI-lower-Adj	CI-upper-Adj
Reference	0.969	0.126	7.693	<0.001	2.636	2.059	3.374	1.000	1.000	1.000
BMI	0.087	0.117	0.748	0.455	1.091	0.868	1.371	0.414	0.329	0.520
Age	−0.003	0.004	−0.726	0.468	0.997	0.989	1.005	0.378	0.375	0.381
BMI*Age	−0.008	0.004	−2.161	0.031	0.992	0.985	0.999	0.376	0.374	0.379
Reference	0.972	0.062	15.761	<0.001	2.643	2.342	2.983	1.000	1.000	1.000
BMI	0.120	0.059	2.043	0.042	1.128	1.005	1.266	0.427	0.380	0.479
Duration of symptoms	−0.022	0.014	−1.622	0.106	0.978	0.953	1.005	0.370	0.360	0.380
BMI*Duration of symptoms	0.003	0.012	0.289	0.772	1.003	0.980	1.027	0.380	0.371	0.389
Reference	0.883	0.030	29.907	<0.001	2.418	2.282	2.562	1.000	1.000	1.000
BMI	0.216	0.059	3.650	<0.001	1.241	1.105	1.393	0.513	0.457	0.576
Ovarian size	−0.058	0.102	−0.568	0.571	0.944	0.773	1.153	0.390	0.320	0.477
BMI*Ovarian size	−0.276	0.080	−3.454	0.001	0.759	0.649	0.887	0.314	0.268	0.367
Reference	0.994	0.067	14.942	<0.001	2.703	2.372	3.079	1.000	1.000	1.000
BMI	0.121	0.028	4.315	<0.001	1.129	1.068	1.192	0.418	0.395	0.441
Family history of PCOS	−0.115	0.062	−1.863	0.063	0.891	0.790	1.006	0.330	0.292	0.372
BMI*Family history of PCOS	−0.031	0.055	−0.563	0.574	0.970	0.871	1.080	0.359	0.322	0.400
Reference	1.058	0.069	15.221	<0.001	2.880	2.513	3.300	1.000	1.000	1.000
BMI	0.059	0.069	0.856	0.393	1.061	0.927	1.215	0.368	0.322	0.422
Ovarian cysts	−0.140	0.051	−2.736	0.007	0.869	0.787	0.961	0.302	0.273	0.334
BMI*Ovarian cysts	−0.003	0.046	−0.055	0.956	0.997	0.911	1.092	0.346	0.316	0.379
Reference	0.463	0.230	2.012	0.045	1.589	1.012	2.495	1.000	1.000	1.000
BMI	−0.155	0.205	−0.759	0.448	0.856	0.573	1.279	0.539	0.361	0.805
HDL	0.010	0.006	1.837	0.067	1.010	0.999	1.021	0.636	0.629	0.643
BMI*HDL	0.000	0.005	0.004	0.997	1.000	0.990	1.010	0.629	0.623	0.635

BMI, Body mass index; HDL, High-density lipoprotein cholesterol; OR, Odds ratio; CI, Confidence interval; *B*, Regression coefficient; Std error, Standard error; OR-Adj, adjusted odds ratio after setting the reference (intercept) to 1; CI-lower-Adj/CI-upper-Adj, lower and upper bounds of the adjusted 95% CI. Statistical method: Multivariate logistic regression analysis with interaction terms.

### ROC curve analysis of the impact of BMI on nutritional intervention in patients of different ages and degrees of ovarian enlargement

**Figure 1:**
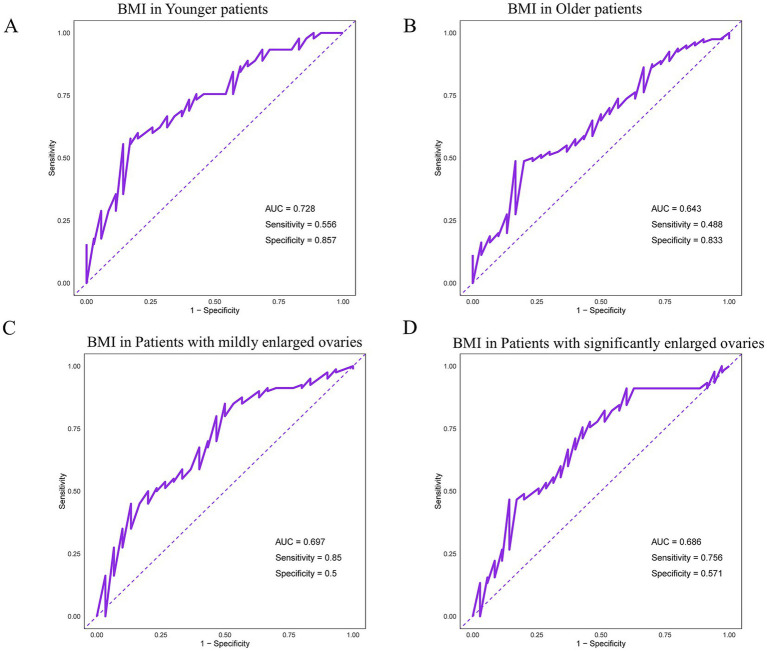
Prediction of nutritional intervention effectiveness by BMI in **(A)** younger patients and **(B)** older patients. BMI predicts the effectiveness of nutritional interventions in patients with **(C)** less ovarian enlargement and **(D)** more ovarian enlargement.

Using ROC curves to further validate the results of the interaction analysis, the area under the curve (AUC) value for BMI was 0.728 in younger patients, while the AUC value was 0.643 in older patients (Figures 1A,B), indicating that BMI had a higher predictive ability for the effectiveness of nutritional interventions in younger patients. The AUC value of BMI was 0.697 in patients with less ovarian enlargement and 0.686 in patients with more ovarian enlargement (Figures 1C,D), indicating that nutritional intervention had significantly different effects on patients with different BMI groups in patients with less ovarian enlargement.

## Discussion

Our results indicate that nutritional intervention had favorable effects on ovulation rate, insulin resistance, lipid profiles, and fasting plasma glucose across PCOS patients in different BMI categories, with the most pronounced therapeutic benefits observed in the obese group. This finding may be attributable to the relatively greater weight loss achieved by obese patients during the intervention and the associated metabolic and endocrine improvements (22). Previous studies have demonstrated that weight reduction can significantly enhance insulin sensitivity and reduce hyperinsulinemia, thereby decreasing insulin-mediated stimulation of ovarian androgen synthesis, alleviating hyperandrogenism, and promoting the restoration of ovulatory function (23). In the obese state, excessive accumulation of adipose tissue may lead to the secretion of various adipokines and inflammatory mediators (such as leptin, adiponectin, and pro-inflammatory cytokines), which interfere with insulin signaling and place the body in a state of chronic low-grade inflammation (24). Nutritional interventions that restricted high-sugar and high-fat foods while increasing dietary fiber and low-glycemic index foods may help reduce adipose tissue–related chronic inflammation and improve the adipokine secretion profile, thereby further enhancing insulin sensitivity. Moreover, improvement in insulin sensitivity may in turn facilitate the recovery of hypothalamic–pituitary–ovarian axis function, normalize gonadotropin secretion patterns, and create a favorable endocrine environment for the restoration of ovulation (25).

One of the highlights of this study was the interaction analysis and further verification using ROC curves. The results showed that nutritional interventions have different effect on young patients with different BMI. In patients above the median age, the impact of BMI on nutritional intervention became less significant, indicating that age plays an important regulatory role in the effectiveness of nutritional intervention. Young patients typically have a more active metabolic system and relatively balanced hormone levels, making them more sensitive to nutritional intervention. Therefore, BMI directly affected the effectiveness of nutritional intervention in young patients. Younger patients with higher BMI were more likely to experience the benefits of weight loss after intervention, such as improving insulin resistance, regulating blood lipids, and restoring hormone balance. This physiological adaptability makes BMI an important influencing factor in young patients. Older patients with higher BMI often have compensatory mechanisms and a degree of drug resistance in their bodies, and even if their diet and weight are changed through nutritional interventions, their bodies may not adapt as quickly or produce significant responses as young people. The older the age, the slower the metabolism, which may reduce the impact of weight changes on health (26). It is noteworthy that the interaction between age and BMI, although statistically significant, had an OR of 0.992, which is close to 1, indicating that an increase of 1 year in age and 1 kg/m^2^ in BMI has a minimal impact on the likelihood of improved prognosis. This may be because the intervention outcomes in PCOS patients are influenced by multiple factors, including basal metabolism, hormone levels, dietary adherence, and lifestyle, while the effect of short-term interventions is also limited by time. From a clinical perspective, the interaction effect of age and BMI should be interpreted with caution and in the context of clinical relevance.

We also found that, in patients with less ovarian enlargement, BMI had a more significant impact on the effectiveness of nutritional interventions, while in patients with more ovarian enlargement, the role of BMI was weakened. This may be because, in PCOS patients with less ovarian enlargement, the damage to their ovarian function was relatively mild (27). Weight gain can exacerbate insulin resistance, thereby affecting hormone secretion and ovulation function of the ovaries. Nutritional intervention can help reduce weight and improve insulin sensitivity. Therefore, in PCOS patients with less ovarian enlargement, changes in BMI had a more significant impact on ovarian function. In PCOS patients with significantly enlarged ovaries, ovarian function was usually more severely impaired (28). Even with weight loss, abnormal structure and function of the ovaries may have limited the effectiveness of nutritional interventions. In other words, patients with significantly enlarged ovaries may not be able to significantly restore normal ovarian function due to structural changes and functional impairments caused by weight loss.

Although the dropout rate in this study was relatively low (approximately 12.6%), the participants who dropped out may have differed from those who completed the intervention in characteristics or adherence, such as age, BMI, lifestyle, or compliance. This could have resulted in the analyzed sample being more likely to respond to the intervention than the originally enrolled population, introducing potential selection bias. Future studies could reduce this bias by implementing stricter follow-up management, adherence monitoring, and using intention-to-treat (ITT) analysis.

This study also has certain limitations. Being a retrospective study, it is subject to potential selection bias and unmeasured confounding factors, such as dietary adherence, physical activity, and socioeconomic status. In addition, the sample size was relatively small, and the study was conducted at a single center. The 3-month intervention period was short, allowing only a preliminary assessment of the short-term effects of nutritional intervention on weight control and metabolic improvements in PCOS patients, but it was insufficient to evaluate the long-term sustainability of outcomes. Therefore, future research could involve prospective, larger-scale randomized controlled trials with longer follow-up periods to validate the findings of this study. All participants in this study were Chinese, which may limit the applicability of the results to other ethnic or regional populations. Future studies should consider including participants of different ethnicities to further validate the generalizability and applicability of these findings. This study used the globally recognized BMI classification, which may underestimate metabolic risk in Asian patients with lower BMI levels. Future studies should adopt the BMI classification adjusted for Asian populations to more accurately reflect the relationship between metabolic abnormalities and intervention responses.

## Conclusion

We retrospectively included 360 cases and grouped them according to different BMI categories to implement nutritional intervention. The results showed that the obese group achieved the best outcomes (increased ovulation rate, decreased HOMA-IR, the largest absolute change in BMI, and the most pronounced improvements in LDL, HDL, and FPG). Furthermore, the interaction analysis indicated that nutritional intervention was most effective in obese patients aged <30 years and with ovarian enlargement <4 cm. This study provides a reference for individualized nutritional intervention in PCOS patients.

## Data Availability

The raw data supporting the conclusions of this article will be made available by the authors, without undue reservation.
