# Supplementary material for: The impact of nutritional intervention on the prognosis of PCOS patients with different BMIs
Source: Front Med (Lausanne). 2026 Mar 11;13:1650724. doi: 10.3389/fmed.2026.1650724 (PMC13013366; doi:10.3389/fmed.2026.1650724)
Supplement: Supplementary file 1 [file Table_1.docx]

| **Supplementary Table. 1 Baseline characteristics analysis of dropout cases** | | | | | |
| --- | --- | --- | --- | --- | --- |
|  | **All Patients (n=52)** | **Normal weight group (n=25)** | **Overweight group (n=15)** | **Obese group (n=12)** | ***p* value** |
| **Age** | 32 (18-42) | 32 (18-41) | 31 (21-42) | 31 (18-42) | 0.811 |
| **Duration of Symptoms (year)** | 5 (0-8) | 5 (0-7) | 5 (0-8) | 4 (0-8) | 0.956 |
| **Menstrual Cycle Regularity** |  |  |  |  | 0.693 |
| Regular | 13 (25%) | 7 (28%) | 4 (26.67%) | 2 (16.67%) |  |
| Irregular | 32 (61.54%) | 16 (64%) | 9 (60%) | 7 (58.33%) |  |
| Absent | 7 (13.46%) | 2 (8%) | 2 (13.33%) | 3 (25%) |  |
